# Meigs syndrome presenting with axillary vein thrombosis and lymphadenopathy: a case report

**DOI:** 10.1186/1752-1947-7-182

**Published:** 2013-07-15

**Authors:** Ridhima Iyer, Jason Chow, Mona El-Bahrawy, Philip Savage

**Affiliations:** 1 Department of Medical Oncology, Imperial College Healthcare NHS Trust, Charing Cross Hospital, Fulham Palace Rd, London W6 8RF, UK; 2 Department of Histopathology, Imperial College London, Hammersmith Hospital, DuCane Road, London W12 0HS, UK

**Keywords:** Ascites, Meigs syndrome, Ovarian cancer, Thrombosis

## Abstract

**Introduction:**

Meigs syndrome is a rare condition, occurring in less than 1% of ovarian tumors and has the characteristic features of a benign ovarian tumor, ascites and a pleural effusion. We present a case of Meigs syndrome in a young patient presenting initially with an axillary vein thrombosis and local lymphadenopathy.

**Case presentation:**

A 28-year-old Caucasian woman presented with a short history of right arm swelling and shortness of breath as a result of an axillary vein thrombosis and pulmonary embolus.

The initial assessment also demonstrated right axillary and subclavian lymphadenopathy, a pleural effusion, ascites and a large ovarian mass. Serum levels of the tumor markers human chorionic gonadotropin and alpha-fetoprotein were normal and the CA-125 level was only moderately elevated.

The combination of thrombosis, lymphadenopathy and an ovarian mass raised the possibility of a disseminated malignancy potentially an epithelial ovarian cancer, a germ cell tumor or an ovarian sex cord-stromal tumor.

Surgery, performed after a short period of anticoagulation, demonstrated a 13.5cm ovarian cellular fibroma of low malignant potential. Postoperatively the patient made an excellent recovery and the ascites, pleural effusion and lymphadenopathy all resolved promptly.

**Conclusions:**

In Meigs syndrome the classical findings of ascites, pleural effusion in combination with an ovarian mass can mimic disseminated malignancy but resolve spontaneously after surgery. In this current case, the patient also had lymphadenopathy and venous thrombosis, two other findings that are frequently associated with malignancy and was acutely unwell at presentation.

It is unclear if the thrombosis and lymphadenopathy were simply coincidental or shared the same etiology as the ascites and pleural effusion. This case indicates that Meigs syndrome may on occasion present with additional findings that can further mimic disseminated malignancy and may lead to diagnostic uncertainty.

## Introduction

Meigs syndrome has a classical triad of features: a benign ovarian tumor combined with ascites and a pleural effusion [1]. The condition is seen in association with less than 1% of ovarian tumors and is very rare in younger women [2].

The clinical presentation is most frequently related to the ascites and generally right-side pleural effusion, while there may also be history of non-specific symptoms such as abdominal discomfort, fatigue, and weight loss and a tumor arising from the pelvis may be palpable.

The investigations in Meigs syndrome usually show normal or only mildly raised serum CA-125 levels and normal human chorionic gonadotropin (hCG) and alpha-fetoprotein (AFP) levels [3,4]. The ascitic fluid can have the characteristics of either an exudate or transudate and there should be no malignant cells in the ascitic fluid cytology [5]. The pathogenesis of the ascites and the pleural effusion are currently unclear. The fluid accumulation may occur due to a relative lack of lymphatic drainage in the large benign tumors leading to pressure lead exudation of interstitial fluid [6] or may be related to the production of vascular endothelial growth factor (VEGF) and fibroblast growth factor (FGF) by the tumor leading to increased local capillary permeability [7].

The management of Meigs syndrome is surgical and the prognosis is excellent with normally prompt resolution of the pleural effusion and ascites postoperatively [8]. Although cases of Meigs syndrome presenting with atypical findings including pericardial effusions [9] have been reported, unusual presentations of Meigs syndrome are rare particularly in younger patients. This current case presented with a pulmonary embolus and distant lymphadenopathy and we are not aware of any other cases presenting in this way.

## Case presentation

A 28-year-old Caucasian woman presented to our Emergency Unit with a short history of progressive shortness of breath and a swollen right arm. She had previously been in good health with no medical history of note but was just recovering from a severe upper respiratory tract infection, which had caused her to be unwell in bed for several days. Aside from the oral contraceptive pill she took no regular medication and had no family medical history of note.

**Figure 1:**
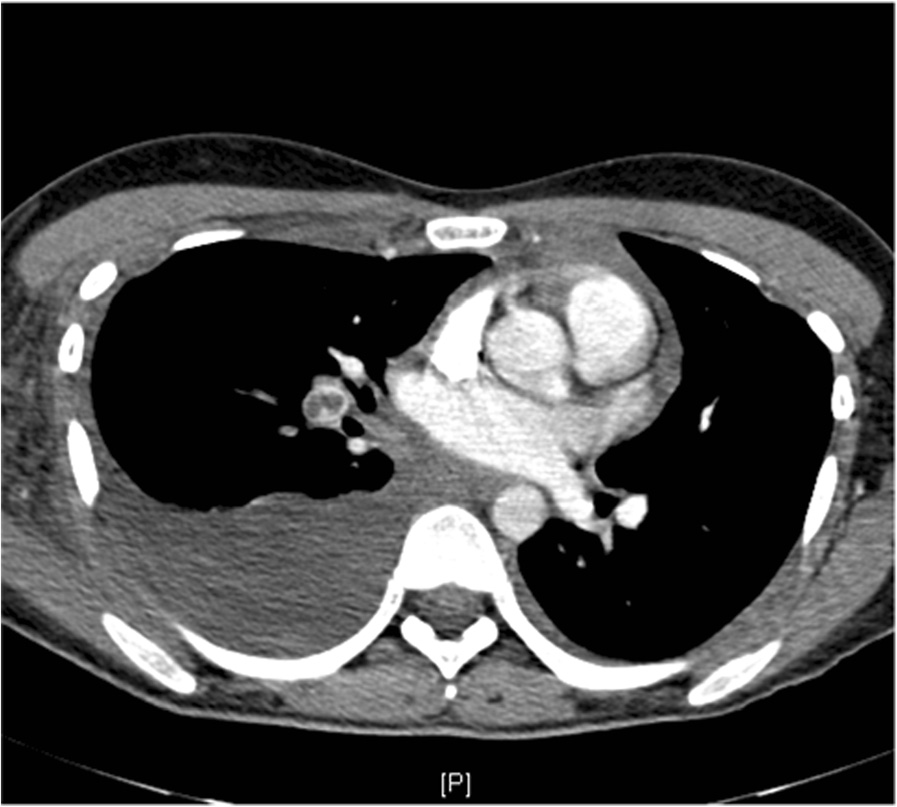
Computed tomography scan of the thorax demonstrating an acute thrombus within the right lower lobe pulmonary artery in keeping with a pulmonary embolus and a large right pleural effusion.

**Figure 2:**
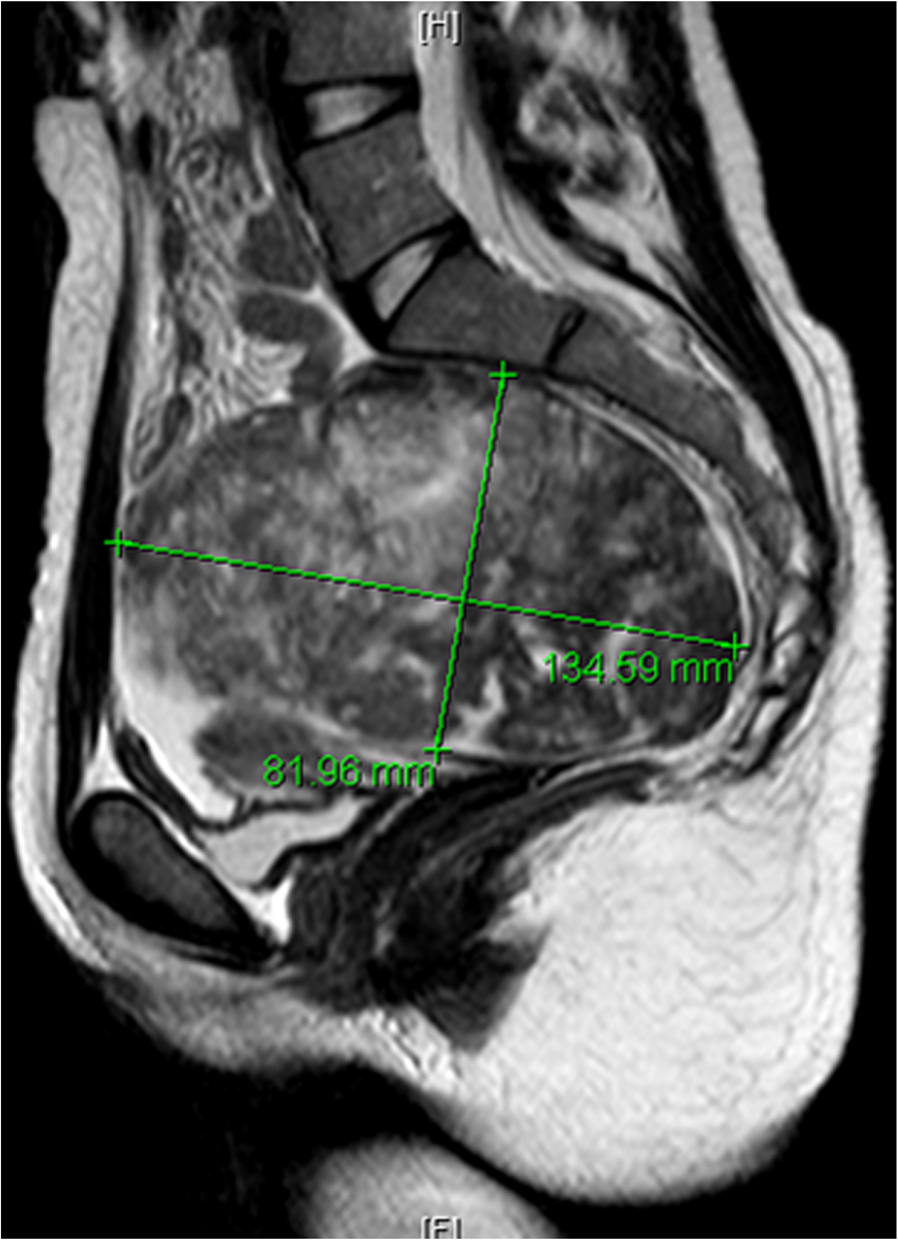
Magnetic resonance imaging scan of the pelvis demonstrating a large heterogeneous mass arising from the left ovary, extending from the sacrum to the abdominal rectus muscle.

Initial investigations with a Doppler ultrasound of her right arm and axilla demonstrated a deep vein thrombosis in the right subclavian vein with extension into the proximal internal jugular vein. A computed tomography scan confirmed the presence of the right axillary vein thrombus and the pulmonary embolus and a large right pleural effusion as shown in Figure 1. Despite these findings, the patient was hemodynamically stable and after review by the vascular surgery team the decision was made to manage with anticoagulation rather than thrombolysis. The initial imaging also demonstrated enlarged lymph nodes, without the normal fatty hilum, in the mediastinum and right supraclavicular fossa. The imaging of the patient’s abdomen and pelvis demonstrated a mixed echogenicity 13.5cm left adnexal mass situated between the sacrum and the anterior abdominal wall shown in Figure 2.

The tumor markers performed at presentation demonstrated normal values for both hCG and AFP whereas the CA-125 level was moderately elevated at 368U/mL (normal range 0 to 35). The initial management centered on the treatment of the pulmonary embolus with anticoagulation and the insertion of a prophylactic inferior vena cava filter. The differential diagnoses at this stage included: epithelial ovarian cancer, an ovarian germ cell tumor, an ovarian sex cord-stromal tumor or an atypical Meigs syndrome.

After a week of anticoagulation treatment, a laparotomy was performed which demonstrated a large left ovarian mass with ascites but no other abnormal findings. The ovarian mass was removed along with a biopsy of the omentum and a fine needle aspirate of the supraclavicular lymph nodes was performed. Postoperatively the patient made an excellent recovery and was well enough to be discharged home 6 days later.

**Figure 3:**
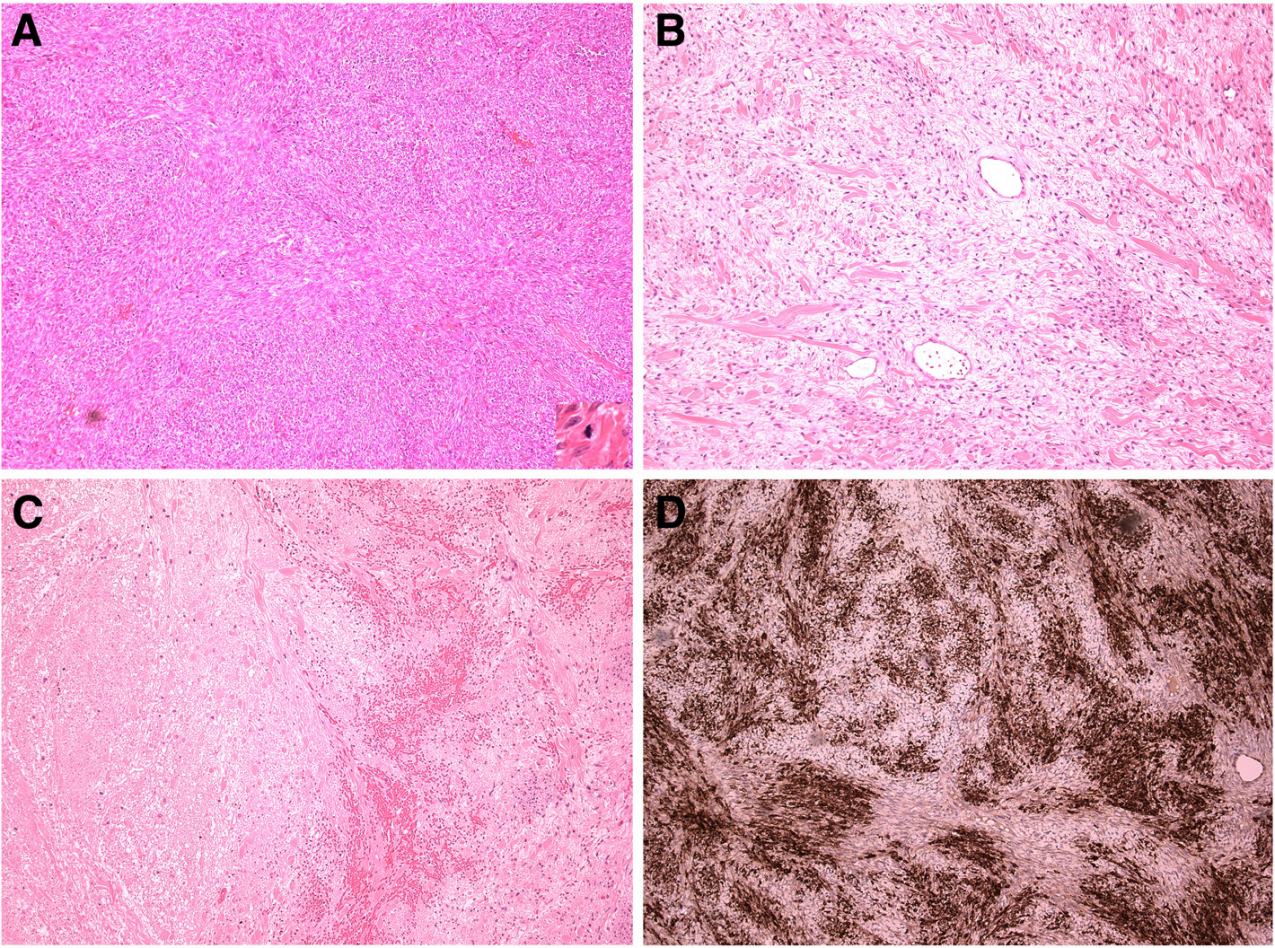
**Histological features of the ovarian cellular fibroma. A**: The tumor cells showed hypercellular areas with mitotic activity (inset) and **B**: hypocellular areas with stromal edema and collagen bundles. **C**: There were areas of hemorrhage and necrosis. **D**: Immunostaining showed strong expression of inhibin. (Magnification A to D = ×100, inset in A = ×400).

On pathological examination the ovarian mass measured 14×8.5×6.5cm and weighed 470g. The outer surface was predominantly smooth with a focus of roughened area, suggesting a site of adhesion to adjacent structures. The cut surface was solid, tan in color and focally hemorrhagic. Histological examination, as shown in Figure 3, demonstrated a spindle cell tumor of variable cellularity with both hypercellular and hypocellular areas. The tumor cells were arranged in sheets and intersecting fascicles with the intervening stroma containing short collagen bundles and areas of necrosis and focal hemorrhage. The cells had oval to spindle nuclei with fine chromatin and inconspicuous nucleoli. No notable cytological atypia was seen. The mitotic activity was variable with most parts showing no mitotic figures while focally mitoses were seen with up to two mitotic figures per 10 high-power fields (HPF).

On immunostaining the tumor cells expressed inhibin, calretenin (focally), progesterone receptor (weak expression in some cells) but were negative for estrogen receptor, CD34, HMB-45, S-100 protein, h-caldesmon, MNF116, epithelial membrane antigen, CAM 5.2 and cytokeratin 5/6. The morphology and immunoprofile were those of cellular fibroma. The omental tissue showed only mesothelial hyperplasia, and no tumor deposits and the ascitic fluid showed no malignant cells. The biopsy from the enlarged supraclavicular lymph nodes showed prominent interstitial reticulum cells, but no evidence of malignancy.

The pathological findings, coupled with the rapid spontaneous resolution of the ascites, pleural effusion and lymphadenopathy postoperatively, confirmed the diagnosis of Meigs syndrome, combined with reactive lymphadenopathy.

The patient was referred to the hematology team for investigation of any clotting tendency. Fortunately, the results of all investigations including Factor V, lupus and anti-cardiolipin screens were negative and routine anticoagulation was continued for 1 year and then discontinued. After 5 years of follow up the patient remains in good health and has recently had a successful pregnancy with a healthy baby girl delivered without complications.

## Discussion

Meigs syndrome was originally described in 1937 and comprises a characteristic triad of features: a benign ovarian tumor, usually a fibroma, combined with ascites and a pleural effusion [1]. Ovarian fibromas are the commonest benign ovarian tumors and account for approximately 4% of all ovarian tumors. However, less than 10% of these cases present in women under the age of 30 years and Meigs syndrome is overall seen in less than 1% of ovarian fibromas [6].

Ovarian fibromas are usually characterized by an indolent clinical presentation and are frequently diagnosed as an incidental finding on imaging with a typical solid hypoechoic but non-vascular appearance on ultrasound and with low T1 and T2 signal with magnetic resonance imaging [10]. The tumor does not produce hCG or AFP and usually has normal or lower CA125 levels compared to those seen in association with more common epithelial ovarian tumors. In ovarian fibromas the CA125 production occurs as a result of peritoneal irritation rather than direct production from the tumor [11].

Although these findings are characteristic of ovarian fibromas, other more complex types of ovarian pathologies including ovarian germ cell tumors, sex cord-stromal tumors and fibrosarcoma of the ovary can also present with similar clinical findings and the diagnosis of Meigs syndrome can only be made after excluding the more serious potential pathologies. Pathologically ovarian fibromas are densely cellular tumors generally light yellow in color with spindle-shaped cells, high levels of intercellular collagen and frequent hemorrhages. The key diagnostic differential is to distinguish the low malignancy potential fibroma from the aggressive malignancy of ovarian fibrosarcoma. Historically this differentiation has been established using a proliferation index with less than three mitoses per 10 HPF linked to benign disease and those with four to 25 mitoses per 10 HPF associated with malignant fibrosarcoma [2]. However, more recently other studies have indicated that some of the more mitotically active tumors but with a cytologically bland appearance also have a favorable prognosis [12].

The pathogenesis of the production of ascitic and pleural fluid in Meigs syndrome is uncertain. It has been postulated that the fluid results from an imbalance between vascular supply and lymphatic drainage in these large tumors, An alternate hypothesis is that the process is inflammatory in nature, with an earlier report demonstrating elevations of several inflammatory molecules and cytokines including VEGF, FGF, interleukin (IL)-1b, IL-6 and IL-8, however, the detailed underlying mechanism is still unclear [7]. Interestingly the preoperative cytokines tested in the patient in this report demonstrated normal values for IL-1b and IL-8 and only modest elevation of the IL-6 and tumor necrosis factor levels.

Meigs syndrome is characterized by a rapid resolution of the ascites and pleural effusion postoperatively and these occurred rapidly in the patient in this study. In keeping with the benign natural history of the syndrome, there have been no further problems and the patient has recently completed a successful pregnancy.

## Conclusions

The diagnosis of Meigs syndrome carries a good prognosis with no significant long-term health issues. In this case we have highlighted that patients with Meigs may present in combination with other clinical features that may further support the usual mimicry of the syndrome of disseminated malignancy. In this case, despite the complex presentation, surgery combined with standard medical management led to a complete resolution of the presenting symptoms.

## Consent

Written informed consent was obtained from the patient for publication of this case report and accompanying images. A copy of the written consent is available for review by the Editor-in-Chief of this journal.

## Competing interests

The authors declare that they have no competing interests.

## Authors’ contributions

RI, JC ME-B and PS were all major contributors in writing the manuscript. All authors read and approved the final manuscript.
